# An In Situ Study to Understand Community Structure of Estuarine Microbes on the Plastisphere

**DOI:** 10.3390/microorganisms10081543

**Published:** 2022-07-29

**Authors:** Ana Sosa, Feng Chen

**Affiliations:** Institute of Marine and Environmental Technology, University of Maryland Center for Environmental Science, Baltimore, MD 21202, USA; asosa@umces.edu

**Keywords:** microplastics, estuary, microbial community, biofilms, plastisphere

## Abstract

Microplastics are defined as pieces of plastic that are smaller than 5 mm and they are now considered one of the most abundant ubiquitous plastic debris. Microbial communities that settle on particles can potentially lead to the transport of pathogenic and harmful bloom-forming species, as well as have an impact on global biogeochemical cycles. However, little is known about the acclimation of microbes to different types of microplastic in the estuarine environment. In this study, 16S ribosomal RNA sequencing and analysis was performed on biofilm samples from three different types of microplastic beads placed in Baltimore’s Inner Harbor. Microbial communities associated with microplastic particles and glass bead control were monitored throughout the 28-day incubation time. A significant taxonomic composition dissimilarity was observed between particles-associated and free-living communities, suggesting a unique microbial adaptation to these biofilms. The polymer types, however, did not significantly influence the microbial community composition. Some families with interesting potential metabolism were identified in the plastisphere samples, including Cyanobacteria, Planctomycetes, Desulfobacteriota, and Firmicutes, leading into speculation of their ecological responses and metabolic roles in the estuarine environment. It is crucial to understand the microorganisms that inhabit plastic debris in estuarine systems and their potential metabolic capacity and how it may differ from its marine counterparts in order to assess their roles in global nutrient cycles and if they have ability to be utilized in bioremediation for plastic pollution.

## 1. Introduction

In today’s world, plastic pollution is a ubiquitous problem. Plastic debris can be found in virtually every environment: from glaciers to sediments and in all bodies of water as well [1]. The physical characteristics that make synthetic polymers ideal for manufacturing, such as durability and solidity, also makes them the perfect growth surface for a wide array of organisms. Debris of all sizes can be found in aquatic ecosystems but there is a particular concern for particles that are smaller than 5 mm, known as microplastics [2]. These particles can be classified as primary or secondary microplastics, with the first being those that were manufactured to be in this size range and the second being those that break down from large pieces of plastic [3,4]. Microplastics can be extremely difficult to quantify, isolate, and characterize and they can be virtually impossible to remove from ecosystems without disturbing them. Research into the environmental impact of microplastics has been an emergent topic in the last decade, especially in sediments and marine systems [5], but insight into their effects in estuaries is not yet sufficient or extensive.

The Chesapeake Bay is the largest estuary in the United States, and includes four rivers: the Susquehanna, the Potomac, the Patapsco, and the Patuxent. In 2011, a study trawled four estuarine tributaries (Patapsco, Rhode, Magothy, and Corsica) of the Chesapeake Bay and found significant concentrations of microplastic particles in a wide range of concentrations and concluded that the locations that had the higher concentration of particles correlated to locations with higher population densities [6]. Studies on microplastics in the surface waters of the Chesapeake Bay have found that polymer particles are present in concentrations from 0.007 to 1.245 particles per m^3^, and that polypropylene (PP) and polyethylene (PE) are the most common types [7].

Microorganisms colonize surfaces and form biofilms in order to protect themselves from grazing, mitigate the competition between species, facilitate horizontal gene transfer, exchange nutrients, and to overall increase their probability of survival [8]. Biofilms can be complex and be conformed of multiple layers of prokaryotes and eukaryotes that make up a constantly adapting community that can interact and become more and more diverse. Biofilms on plastic particles are no exception, and microplastics provide further advantages for microbes as they can subsist for decades in aquatic environments and the roughness and enhanced half-life of these particles contribute to making them the ideal candidates for colonization. In 2013 a study found heterotrophic bacteria, filamentous cyanobacteria, and some eukaryotes such as diatoms attached to the surface of microplastic particles and named the biofilms the “plastisphere” [9]. The plastisphere has a greater evenness compared to the communities of the water around them that included many rare and undescribed species [9]. Physical factors such as water flow and UV light exposure can significantly increase the roughness of particles by creating cracks and holes and/or oxidation of their hydrophobic surface [9]. Microcosm studies have shown that the biofilm formation on microplastic particles is fast and total colonization can take anywhere from a couple of days to a couple of weeks [10]. Some of the factors that determine the rate of colonization and the species composition of the biofilms are the chemical and morphological characteristics of the plastic particles, seasonality, geographical location [11], and other environmental conditions such as salinity, temperature, hydrology, and oxygen availability [9,12].

Recently, the total biomass of the oceanic plastisphere has been approximated to represent 0.01–0.2% of the total microbial biomass of the ocean [13]. The formation of biofilms can cause microplastics that would normally float on the water’s surface to become biofouled and sink. They can be accumulated in sediments or even be ingested and incorporated into the food web as part of the microbial loop [14]. Studying the role of microplastics in the cycling of the elements will be crucial to understanding the effects of plastic debris [15] especially considering the fact that growth limiting elements such as nitrogen and phosphorus can become more available to microbes growing on microplastic surfaces than those in oligotrophic waters [13]. Studies into biofilm formation in controlled environmental conditions have demonstrated that bacteria can grow selectively on plastic substrates, due to their hydrophobicity, and the biofilms can be different to those that form on other naturally occurring particles such as glass and cellulose [16]. 

In this study, three different types of polymers (polypropylene, polylactic acid, and polystyrene) were introduced to the water of Baltimore Inner Harbor inside of mesh containers made of stainless steel, alongside an inert control of glass microbeads. Subsamples were collected from each container and surrounding water at different time points over a 28-day incubation period. Microbial community structure was analyzed by sequencing the 16S ribosomal RNA gene. The objective is to explore if the biofilm microbial community on microplastic particles differs from the microbial community of the surrounding waters, and if there are any significant differences in microbial colonization of chemically different polymers and a non-plastic control (glass). It is expected that microbial biofilm communities developed on microplastics will be different from free-living microbial communities in the surrounding water, and the chemical composition and physical properties of the substances making up the microparticles will be a determining factor for the microbial community composition attached. 

## 2. Materials and Methods

### 2.1. Selection of Microplastics

**Figure 1:**
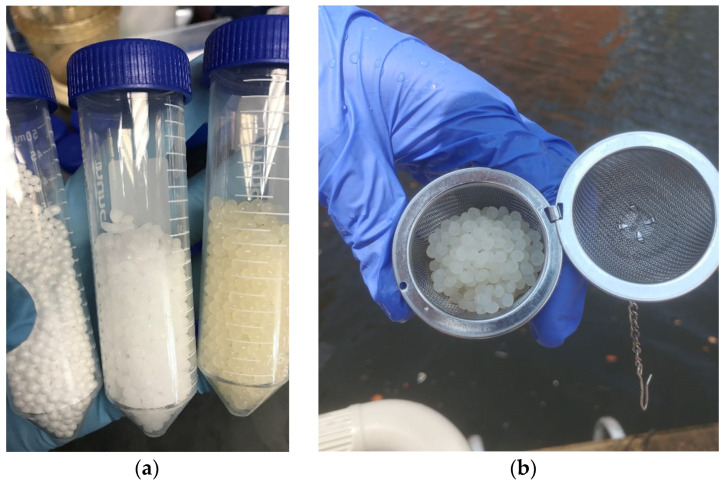
(**a**) Microplastic beads composed of polystyrene, polypropylene, and polylactic acid, respectively. (**b**) Stainless steel mesh container with polylactic acid microbeads.

PE and PP make up the largest portion of the plastic debris in the Chesapeake Bay water (32% and 13%, respectively) [7]. PP was chosen for this study as a solid polymer that is less dense than the estuary’s water and would float on the surface, polystyrene (PS) was chosen as an expanded plastic with a large surface area that would also float on the surface, and polylactic acid (PLA) was chosen as a biodegradable polymer option that does not float to evaluate the differences between these characteristics (Figure 1). The glass beads were selected as an inert surface control to evaluate how a non-organic substrate would be colonized by the same microbial communities.

### 2.2. Preparation and Sterilization of Beads

The three different microplastic spheres composed of pure PP (PollyPlastics^TM^, Farmington Hills, MI, USA), PS (Fairfield^®^, Fairfield, CT, USA), and PLA (3DXTech©, Grand Rapids, MI, USA) were used for this experiment. Glass spheres with a 4 mm diameter (BioSpec, OK, USA) were used as a no-plastic control. They were sterilized prior to being introduced into the water by soaking them in 70% *v*/*v* ethanol overnight and subsequently rinsed with sterile deionized water. Plastic and glass beads were placed in stainless steel mesh containers which were also sterilized before use in the same manner. Stainless steel mesh containers were chosen because they allow free water flow. 

### 2.3. In Situ Incubation

The stainless steel mesh containers containing the microbeads (2–4 mm) were introduced into the Baltimore’s Inner Harbor sampling site (39°17′11.05′′ N, 76°36′22.77′′ W) on 5 September 2020 and allowed to be colonized for 28 days at a depth of one meter under the surface. Time point samples were taken on days 3, 7, 14, 21, and 28 after preliminary experiments showed the most variation within these timepoints. 

### 2.4. Subsampling for Microbial Community Analysis

For each sampling date, 30 microbeads were pooled together to constitute a sample, taken with sterile forceps and in duplicate, one for processing and one for storage and archiving. Microbeads were rinsed using Phosphate-Buffered Saline (PBS) buffer and stored in sterile 2 mL microcentrifuge tubes. Surrounding water samples were taken using a sterile 1 L glass bottle and 100 mL of the sample were filtered on a reusable filter tower (ThermoScientific™, Waltham, MA, USA) with a filter with a 0.2 µm pore size for the retention of microorganisms. 

### 2.5. 16S Sequencing and Bioinformatic Pipeline

DNA from biofilms for both experiments was extracted using the IBI Soil DNA Extraction Kit (IBI Scientific, Dubuque, IA, USA). Water samples were processed using the PowerWater DNA Isolation Kit (QIAGEN, Germantown, MD, USA). Polymerase Chain Reaction (PCR) primers of the 16S ribosomal RNA 515F and 806R target the V4 region (F-GTGYCAGCMGCCGCGGTAA, R-GGACTACNVGGGTWTCTAAT) and have an annealing temperature of 50 °C. The cycle used started with a 94 °C step for 3 min, and then 35 repetitions of 25 s at 94 °C, 60 s at 50 °C, and 90 s at 72 °C. The PCR cycle was finished with a 10 min 94 °C final extension stage. PCR amplicons were sequenced using MiSeq sequencer (Illumina, CA, USA) at the BioAnalytical Services Laboratory at the Institute of Marine and Environmental Technology. Obtained reads were paired and trimmed for quality using CLC Genomic Workbench 8 with default parameters and put through the QIIME bioinformatic pipeline for microbial community analysis utilizing the SILVA 132 taxonomy database (de.NBI). QIIME 2 was used to filter the raw reads for sequencing quality, denoise with the dada2 plug-in, and to pick operational taxonomic units (OTU) [17]. OTUs were filtered by a minimum sequence number of 10 and subsequently classified by taxonomy using the Naive Bayes classifier trained for the V4 region of the 16S rRNA genes (515F/806R primers) and organized by abundance.

### 2.6. Diversity Measures and PERMANOVA

An alpha rarefaction curve was generated using QIIME 2 based on the observed features (OTU) and the sequencing depth. Phylogenetic metrics and statistical analysis using PERMANOVA was performed in the form of pairwise tests to determine group significance. Principal component analyses (PCoA) using the weighted and unweighted unifrac diversity measures were run to determine community dissimilarity in beta diversity, unweighted to account for presence and absence of OTUs and weighted to include the abundance of each group. These tests were run on a Python environment by using the pertinent QIIME 2 pipeline commands and with the default parameters of the pipeline [17].

### 2.7. Ecological Interpretation of 16S Marker Data

OTU tables with the corresponding taxonomy assigned were then compared to the FAPROTAX [18] database. This database maps the previously known OTUs to ecologically relevant metabolic functions of known and cultured species that are closely related. The collapse.py command in Python collapses the OTU tables generated by QIIME 2 and converts them into a profile describing the functional group and its abundance in known taxonomic units in the database. This results in an analysis of the metabolic or relevant ecological functions of the described microbial communities from the 16S rRNA gene amplicon characterization using a reference database based on the literature of cultured microbial representatives of genera or species.

### 2.8. Hydrological Data

**Figure 2:**
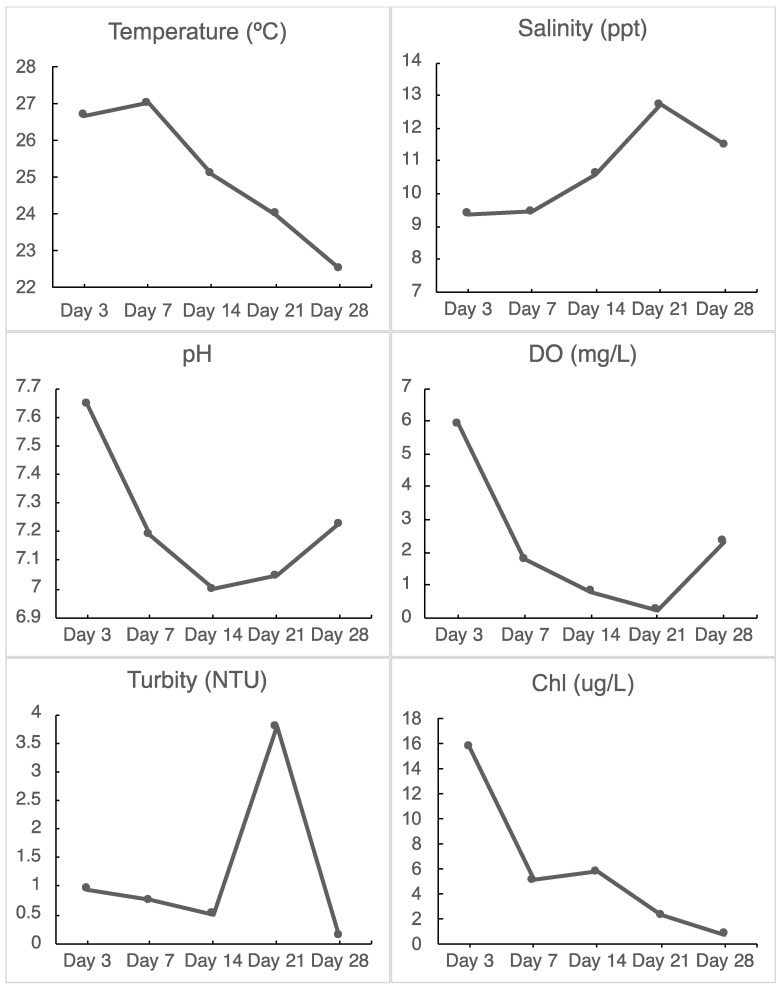
The hydrological data obtained from the Inner Harbor monitoring site of DNR. The site is located at the National Aquarium, about 200 m from the study site. Each bullet represents the average of all measurements of that day.

Water quality data was downloaded from the Department of Natural Resources’ (DNR) Eyes on the Bay project (http://eyesonthebay.dnr.maryland.gov/, (accessed on 10 July 2021)) which has a monitoring station on the east side of the National Aquarium and within 200 m of the sampling site. This station takes a snapshot of water quality every 15 min continuously and measures pH, temperature, dissolved oxygen, salinity, turbidity, and chlorophyll concentration. Data for the pertinent dates (Figure 2) September and early October 2020 were accessed on the DNR website and analyzed to obtain the average conditions for the sampling days.

## 3. Results

A total of 25 subsamples were collected during the in situ incubation period (Table 1). Five subsamples were taken at each time point from the treatments and controls.

### 3.1. Hydrological Data

During the in situ incubation period, salinity fluctuated between 9.3 and 12.7 ppt, and temperature decreased gradually from 27 °C on day 7 to 22.5 °C on day 28 (Figure 2). The concentration of chlorophyll a decreased dramatically from 15.7 to 0.72 µg/L, and the level of dissolved oxygen also decreased markedly (from 5.89 to 0.23 mg/L). It is noticeable that the Inner Harbor water was in the hypoxic condition during the in situ study period and was almost anoxic on day 21. In addition, pH also fell during this period (5 September to 2 October 2020) (Figure 2).

### 3.2. DNA Sequencing and Bioinformatic Pipeline

DNA extracted from the 25 subsamples (Table 2) were sequenced. A summary of the resulting raw reads from the Illumina sequencing of samples is shown in Table 2. Reads were filtered using QIIME2 and the OTUs were clustered and enumerated through the same pipeline. Raw reads were in a range of 16,525–89,012 and the number of OTUs in a range of 12,750–51,068. Generally, it appears that samples from PP, PS, and PLA have the largest number of raw reads and OTUs, with a few exceptions such as the TPS102 sample. The relationship between number of raw reads and final picked OTUs is overall proportional. 

### 3.3. Microbial Community Analysis

**Figure 3:**
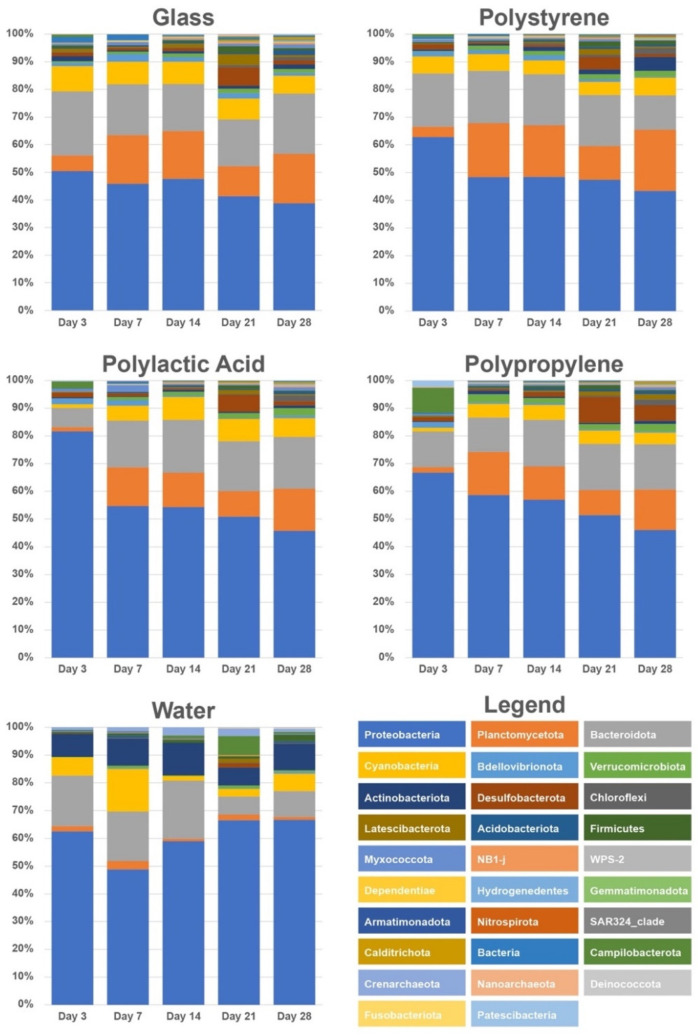
Relative abundance of major bacterial and archaeal lineages at the phylum level for microbial communities. The relative abundance was analyzed based on the 16S ribosomal RNA gene sequences obtained on days 3, 7, 14, 21, and 28.

The change in microbial community at the phylum level in different microplastics, glass beads, and surrounding water during the 28-day incubation period is shown in Figure 3. The community composition of all samples at the class and family level are shown in supplemental information (Figures S1 and S2, respectively). The composition of microbial communities on microplastics and glass beads appeared to be similar to each other from day 3 to day 28 (Figure 3). In contrast, the microbial community in the surrounding water maintained its own populations distinguishable from the biofilm microbial communities (Figure 3). For the biofilm community, phylum Planctomycetes increased from low relative abundance on day 3 to ca. 10–20% for the rest of the incubation time (Figure 3). Such a trend was not seen in the surrounding water (Figure 3). 

It appears that more Proteobacteria colonized on microplastics on day 3 compared to glass beads (Figure 3). On day 3, Proteobacteria made up ca. 82, 66, and 66% of microbial communities of PLA, PP, and PS, respectively, while glass beads contained ca. 50% of Proteobacteria. From day 3 to day 28, the relative abundance of Proteobacteria decreased gradually in all biofilm communities (PP, PS, PLA, and glass beads), but they were still the most abundant microbial group (>40% for all microplastics) at the end of incubation (Figure 3). Bacteriodota appeared to be relatively stable (15–20% in most cases) throughout the incubation period. The abundance of phylum Cyanobacteria either stayed stable (Glass, Polystyrene) or slightly increased (PLA, PP) in all particle-associated particles, whereas it showed a decrease in water communities on days 14 and 21 (Figure 3). Interestingly, the relative abundance of Desulfobacterota increased on all biofilms (PP, PS, PLA, and glass beads) on day 21, and increased somewhat in the water sample on day 21 (Figure 3). Water communities showed a significantly higher abundance of the Actinobacteriota phylum in all time points, as well as a higher abundance of the phylum Crenarchaeota. 

### 3.4. Diversity Measures and PERMANOVA

Alpha rarefaction curves of all material types showed feature count plateauing at around 2000 sequencing depth, suggesting the diversity of the communities is captured at this depth and based on this the p sampling depth was selected as 2000 to determine how many sequences will be randomly subsampled for the following diversity measures.

**Figure 4:**
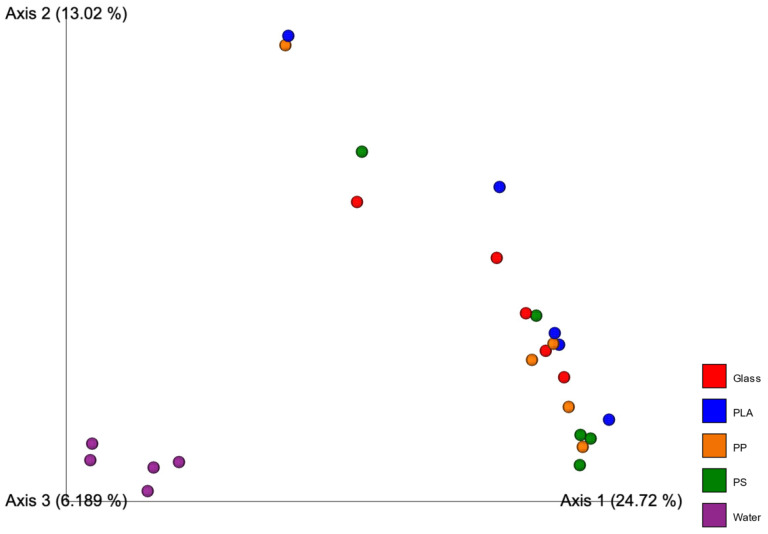
PCoA plots showing the dissimilarity of samples grouped by material they were extracted from. Beta diversity measures show the unweighted unifrac distance.

**Figure 5:**
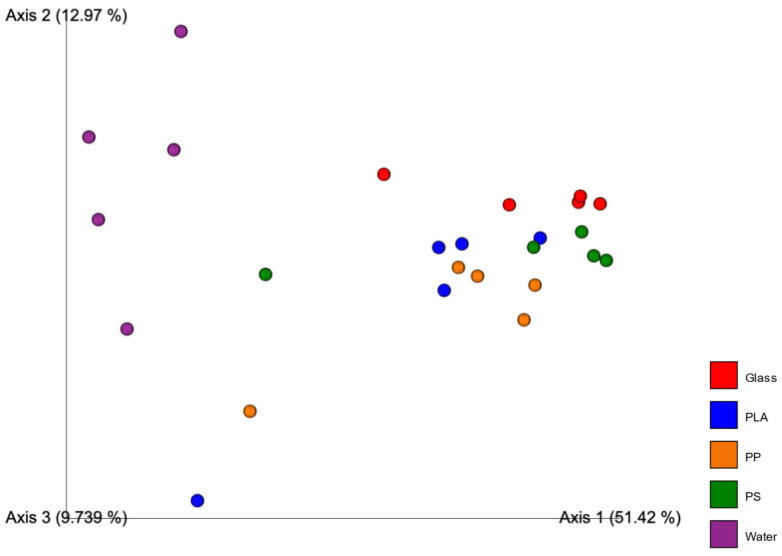
PCoA plots showing the dissimilarity of samples grouped by material they were extracted from. Beta diversity measures show the weighted unifrac distance.

Statistical analysis using pairwise PERMANOVA tests is positive for dissimilarity if the *p*-value is <0.05 to <0.05 (Table 2). Beta diversity represented by PCA plots using unweighted unifrac (Figure 4) and weighted unifrac (Figure 5) distance show the water samples being isolated as a group in all instances. Unweighted unifrac distance analysis does not consider abundance of the OTUs and is a phylogeny-including taxonomic measure. Weighted unifrac distance, on the other hand, considers the abundance of taxonomic units. This provides two perspectives, one that gives weight to the most abundant taxa and one that gives equal weight to abundant and rare taxa.

In both weighted and unweighted analyses, water groups together and separately from all the other particle-associated communities. There is no noticeable clustering of plastic materials separately from the glass inert control in either of the diversity measures.

PERMANOVA analysis confirmed the statistical significance of this dissimilarity. The *p*-values for the comparison between sampling groups showed dissimilarity exclusively when particle-associated communities were compared to water communities (glass–water = 0.01, PLA–water = 0.01, PP–water = 0.006, PS–water = 0.009). Every other *p*-value in the PERMANOVA analysis was larger than the 0.05 cutoff for dissimilarity (Table 2). 

### 3.5. Ecological Interpretation of 16S Marker Data

**Figure 6:**
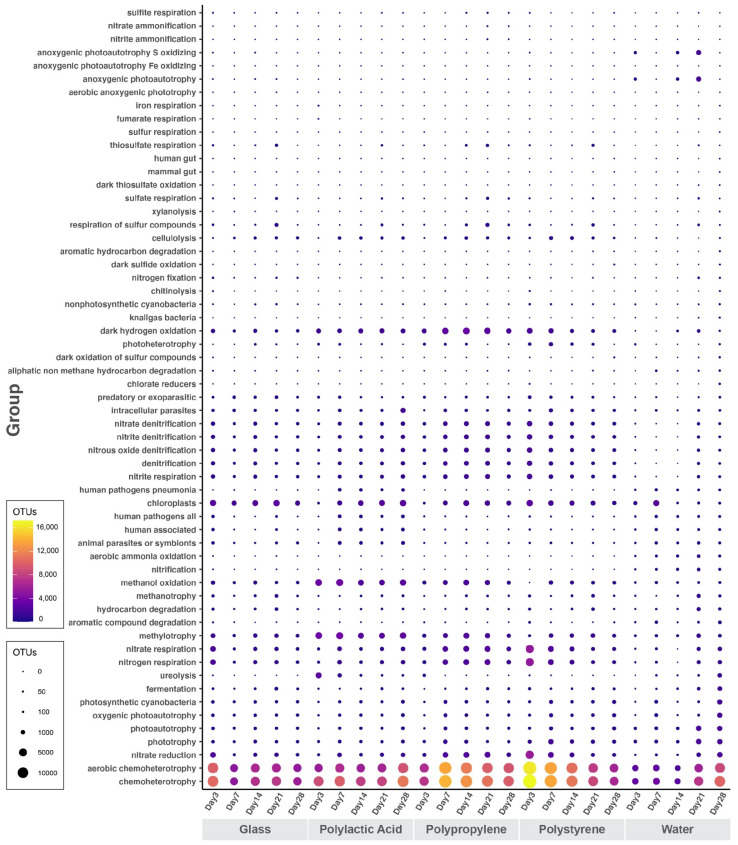
Ecologically relevant function abundance per sample. Bubbles represent the number of OTUs that have been classified by FAPROTAX as a functional group in each sample.

The most abundant potential metabolic phenotype in all OTUs from all samples is chemoheterotrophy (Figure 6). These two are closely followed by phenotypes that are related to phototrophy, photoautotrophy, and lastly by photosynthetic cyanobacteria, with a similar abundance in all samples but slightly higher for all water samples. However, all particle-associated samples contain more chloroplast-containing phenotypes than the surrounding water. All nitrogen metabolism phenotypes are highly abundant in all biofilm samples, especially for PP (Figure 6). This is especially noticeable for nitrate and nitrite denitrification, nitrite respiration, nitrogen respiration, and denitrification. Methylotrophy and methanol oxidation are more abundant in PLA compared to the remaining samples (Figure 6). Dark hydrogen oxidation in PP is more abundant compared to the other samples.

There is no significant increase in OTUs that have been related to plastic degradation in any of the samples, but there is, however, a uniform abundance of hydrocarbon degradation potential metabolism OTUs. Additionally, OTUs related to human pathogenesis (general, pneumonia) seem to be abundant in communities from PLA samples and in those from water samples (Figure 6). Potentially predatory, intracellular parasitic or exoparasitic OTUs are more abundant in all particle-associated communities but animal parasites or symbionts are equally abundant among all samples.

## 4. Discussion

### 4.1. Low Dissolved Oxygen in the Inner Harbor during the Incubation Period

The hydrological data showed that the water’s environmental conditions changed rapidly during the in situ incubation study. Although we do not know how bacterial and algal abundance changed during this period, we speculate that the rapid decrease in chlorophyll a was caused by biomass reduction or die off of phytoplankton. Colder temperatures in the later period of incubation might result in the decrease in phytoplankton abundance. When phytoplankton die, they release a large quantity of organic matter which support massive growth of heterotrophic bacteria. Respiration of organic matter by bacteria consumes oxygen and quickly lowers dissolved oxygen in the harbor. It is likely that the hypoxic/anoxic status was formed due to massive consumption of phytoplankton-released organic matter by bacteria. Meanwhile, bacterial respiration also releases CO_2_, which may contribute to the lower pH observed in the later stage of incubation. It is unclear why the water turbidity increased on day 21. Understanding the change in water quality during the incubation time is an important step towards comprehensive analysis of microbial community on the plastisphere. 

### 4.2. Planctomycetes Thrived on Biofilms

It is noticeable that Planctomycetes became more abundant after day 7 on all microplastic particles and glass beads (Figure 3) and remained stable until the final timepoint of the in situ incubation. Planctomycetes is one of the most commonly reported phyla in marine plastics in a wide range of locations [19]. In this study, Planctomycetes made up ca. 15–20% of microbial communities from day 7 to the end of experiment (day 28). For most of the incubation time, the local water experienced the hypoxic and anoxic condition (Figure 2), and the low-oxygen condition is preferable to Planctomycetes. Fuerst et al. [20] reported that Planctomycetes generally thrive in an attached lifestyle, especially in low oxygen conditions, as they are able to oxidize ammonia to dinitrogen without oxygen. Planctomycetes have been previously found in sediments from Baltimore’s Inner Harbor and their ammonia aerobic oxidation (anammox) activity has been characterized in an effort to understand how an increase in nitrogen due to urban activity and the use of fertilizers has affected the nitrogen cycle in the Chesapeake Bay water [21,22].

### 4.3. Desulfobacteriota Became Abundant on Day 21

The abundance of Desulfobacteriota increased in all samples on day 21 of the incubation experiment and then decreased to the previous levels for the day 28 sampling timepoint (Figure 3). Hydrological data show a decrease in dissolved oxygen for the 21 day timepoint with a drop to 0.23 mg/mL (Figure 2). Metagenomic studies have found a high abundance of mercury methylation genes in Desulfobacteria species that are commonly found in low-oxygen waters of the Baltic Sea and found that these genes were found in higher relative quantities in marine-particle-associated communities than those in free-living communities [22]. These bacteria have been found in oxygen-deficient zones, including both anoxic and hypoxic waters.

### 4.4. Succession of Biofilm Communities

We found that initial colonization could be proven from the day 3 samples and that the number of days of incubation was not proportional to the number of raw reads or classified OTUs obtained (Table 1), as some of the samples with the highest number of reads are from the earliest sampling timepoints (i.e., TPS97). The incubation time was limited to 28 days for a number of factors including the overall stabilization of microbial communities within four weeks and the limited time to conduct experiments due to the 2020 global pandemic. In all particle-associated samples, there was a decrease in proteobacteria after the initial colonization of about 5–25% from day 3 to 7 and it was followed by further decrease throughout the 28-day incubation. The water samples showed the same initial decrease from day 3 to day 7 but slowly recovered during the rest of the timepoints to an even higher abundance on day 28 than on day 3. This suggests that after the initial colonization period in the first few days where Proteobacteria usually dominate, the rest of the members of the particle-associated community has a chance to increase in abundance. The initial colonization patterns described by studies looking into the early stages of marine microbial plastic biofilms is consistent with the dominating classes of the particle-associated communities being Gammaproteobacteria and Alphaproteobacteria [19,23] in the first two sampling days. After these initial stages, the composition of Proteobacteria in all plastic and glass samples seemed to stabilize (Figure 3).

Studies into the successive colonization of plastic pollution in marine ecosystems have shown that once microbes colonize a surface and the biofilm becomes mature, the community stabilizes and less changes in composition are perceivable [23]. Bacterial colonization of any surface can happen extremely rapidly in most environments [24] and biofilm formation on polymers has been reported to happen as quickly as in a few hours to a few days [10]. Differences in community structure between days 3 and 7 could be explained by the different environmental conditions they were exposed to, as these conditions started changing within this time period. There was a decrease in DO, pH, turbidity, and *chl a* concentration and a slight increase in temperature and salinity (Figure 2). Samples from polymers in all dates of this in situ experiment show a higher percentage of cyanobacterial species, indicating that photosynthetic species are attaching and persisting on these particles. In the water and glass communities, however, there was a decrease in the abundance of Cyanobacteria (Figure 3) consistent with the overall decrease in chlorophyll a in the sampling site (Figure 2). This could potentially affect nutrients available in the water and general biogeochemical cycles. Several studies have found photosynthetic species in the plastisphere, including prokaryotes dominated by cyanobacteria [9,12,25]. 

Interestingly, the abundance of Actinobacteria was 10–15% higher in water communities than in particle-associated communities, directly contradicting studies in which Actinobacteria thrive in the plastisphere and increase in abundance after incubation on polymers [25,26]. Actinobacteria are known to play a part in the decomposition of organic materials such as cellulose and chitin and thus in the carbon cycle [26,27], but marine Actinomycetes have also been characterized as mainly free-living bacteria [27]. The abundance of Crenarchaeota in water samples being so high (2–5%) relative to that (0–2%) of the polymer samples is consistent with studies of the marine plastisphere in the Mediterranean Sea, in which all types of archaea were found in low abundances in all plastic related samples [28]. It is possible that the availability of sulfur in the plastisphere is too low for archaeal species to grow and develop and this may be the limiting factor for Crenarchaeota in these biofilms [29,30].

### 4.5. Diversity Measures and PERMANOVA

The PERMANOVA analysis showed that there was no significant difference between all particle-associated groups. This result is consistent with a previous study where chemical composition of the surface was a minor factor in determining the composition of the biofilm [19]. Interestingly, there were no significant differences between the microplastic microbead samples and the inert glass beads control, and this directly contradicts previous findings in which naturally occurring substances were colonized selectively by microorganisms [16], but supports the previously mentioned meta-analysis results regarding naturally occurring particles (wood, cellulose, glass) and their communities being statistically similar to those on polymers [19]. These statistical results support our observations of the community structure based on relative abundance of phyla (Figure 3) in which water communities appear to be distinguishable from all particle-associated communities, which are similar across timepoint samples (Figure 3).

### 4.6. Ecological Interpretation of 16S Marker Data

The bubble plot generated with the FAPROTAX database shows that the most abundant function in all OTUs of all samples is chemoheterotrophy (Figure 6), suggesting that there is an abundance of bacteria that are able to use metabolic products as nutrient sources and perform nutrient regeneration in the communities [31]. Functional families related to prototrophy and photoheterotrophy show high abundance in all samples including water, but photosynthetic cyanobacteria appear more abundant in water communities (Figure 6). This directly contradicts our findings from the 16S rRNA gene community analysis, in which cyanobacteria proved more abundant in polymer and glass samples over surrounding water samples (Figure 3). This discrepancy may be due to FAPROTAX being a uniquely predictive tool and not an actual metabolic descriptor. 

There is some evidence of metabolisms that confer bacteria advantages in low oxygen or anoxic conditions, as many of the identified functions are related to nitrogen metabolism, methane metabolism, and even dark hydrogen metabolism (Figure 6). All these functional families appear to be more abundant in the particle-associated communities (Figure 6) of glass, PLA, PS, and PP. This suggests that the plastispheres attaching and forming on these particles are able to adapt to a low-oxygen or anoxic environment and survive even in the deepest layer of the biofilms where oxygen availability may be low to none (He et al., 2018). 

Microbial OTUs with potentially pathogenic functions appear to be the highest in communities from PLA biofilms and in water, contradicting the theory that pathogens can accumulate in large quantities on plastic biofilms and increase in abundance relative to their abundance in the surrounding water of aquatic environments [9,32]. The higher abundance of exoparasites and intracellular parasites in all samples of particle-associated communities, however, supports this theory.

## 5. Conclusions

To the best of the author’s knowledge, this is the first study looking into the microbial communities attaching to microplastic particles in the Chesapeake Bay estuary. We found that formation of biofilms on particles happened rapidly and was overall dominated by the phyla Proteobacteria. After the initial colonization period, other phyla in the community were able to grow and increased in abundance. Consistent with our initial hypotheses, the microbial communities of the biofilms in this in situ study were found to be significantly different to those in the surrounding water. Understanding how chemically different polymers are colonized in estuarine environments is crucial to elucidating the environmental impacts of plastic pollution. The pollution of our waterways is ubiquitous; future work will have to focus on the interactions of these pollutants with all organisms in the ecosystem and it will have to define the extent of its impact on nutrient cycling, transportation of harmful species, and primary production.

## Figures and Tables

**Table 1:** Number of raw reads, number of reads after filtration, and total number of OTUs per sample.

Material	Date	Raw Reads	Filtered Reads	Total OTUs
PP	September 18	89,012	87,180	51,068
PS	September 7	86,722	84,806	52,429
PP	September 11	81,232	79,914	45,136
PLA	October 2	81,198	79,646	49,963
PLA	September 7	79,838	78,042	20,296
PLA	September 11	71,672	70,183	34,545
PP	September 25	71,366	70,247	44,202
PP	September 7	70,822	69,493	22,443
PP	October 2	70,623	69,284	45,136
PS	September 11	66,111	64,789	44,677
PS	September 18	58,716	57,441	44,557
PLA	September 25	58,648	57,554	35,586
Glass	September 7	56,307	55,149	34,308
PLA	September 18	56,272	55,293	29,932
Glass	September 25	56,230	55,255	44,396
PS	September 25	55,547	54,522	42,135
Water	September 25	53,544	52,547	27,485
Glass	September 18	48,332	47,495	36,619
Water	October 2	43,913	43,030	31,958
Water	October 2	42,485	41,687	33,393
PS	October 2	37,252	36,368	28,003
Glass	September 11	31,910	31,349	24,459
Water	September 11	30,615	29,882	20,690
Water	September 7	20,689	20,241	16,192
Water	September 18	16,525	16,251	12,750

**Table 2:** Group significance PERMANOVA in a pairwise analysis.

Group 1	Group 2	Sample Size	Permutations	Pseudo-F	*p*-Value	q-Value
Glass	PLA	10	999	1.3036273	0.138	0.18125
Glass	PP	10	999	1.4050173	0.088	0.176
Glass	PS	10	999	1.4156289	0.115	0.18125
Glass	Water	10	999	4.6538861	0.01	0.025
PLA	PP	10	999	0.8238911	0.492	0.492
PLA	PS	10	999	1.3589694	0.145	0.18125
PLA	Water	10	999	4.5207674	0.01	0.025
PP	PS	10	999	1.0297202	0.448	0.492
PP	Water	10	999	4.1069582	0.006	0.025
PS	Water	10	999	4.7291459	0.009	0.025

## Data Availability

The data presented in this study are openly available in the Sequence Read Archive at https://www.ncbi.nlm.nih.gov/sra, (accessed on 11 January 2022), reference number PRJNA796384.

## Supplementary Materials

The following supporting information can be downloaded at: https://www.mdpi.com/article/10.3390/microorganisms10081543/s1, Figure S1: Relative abundance of major bacterial and archaeal lineages at the class level. The relative abundance was analyzed based on the 16S ribosomal RNA gene sequences obtained on day 3, 7, 14, 21 and 28; Figure S2: Relative abundance of major bacterial and archaeal lineages at the family level. The relative abundance was analyzed based on the 16S ribosomal RNA gene sequences obtained on day 3, 7, 14, 21 and 28.
