# Supplementary material for: An In Situ Study to Understand Community Structure of Estuarine Microbes on the Plastisphere
Source: Microorganisms. 2022 Jul 29;10(8):1543. doi: 10.3390/microorganisms10081543 (PMC9415314; doi:10.3390/microorganisms10081543)
Supplement: Supplementary file 1 [file microorganisms-10-01543-s001.zip › microorganisms-1797024-supplementary.pdf]

microorganisms

Supplemental materials

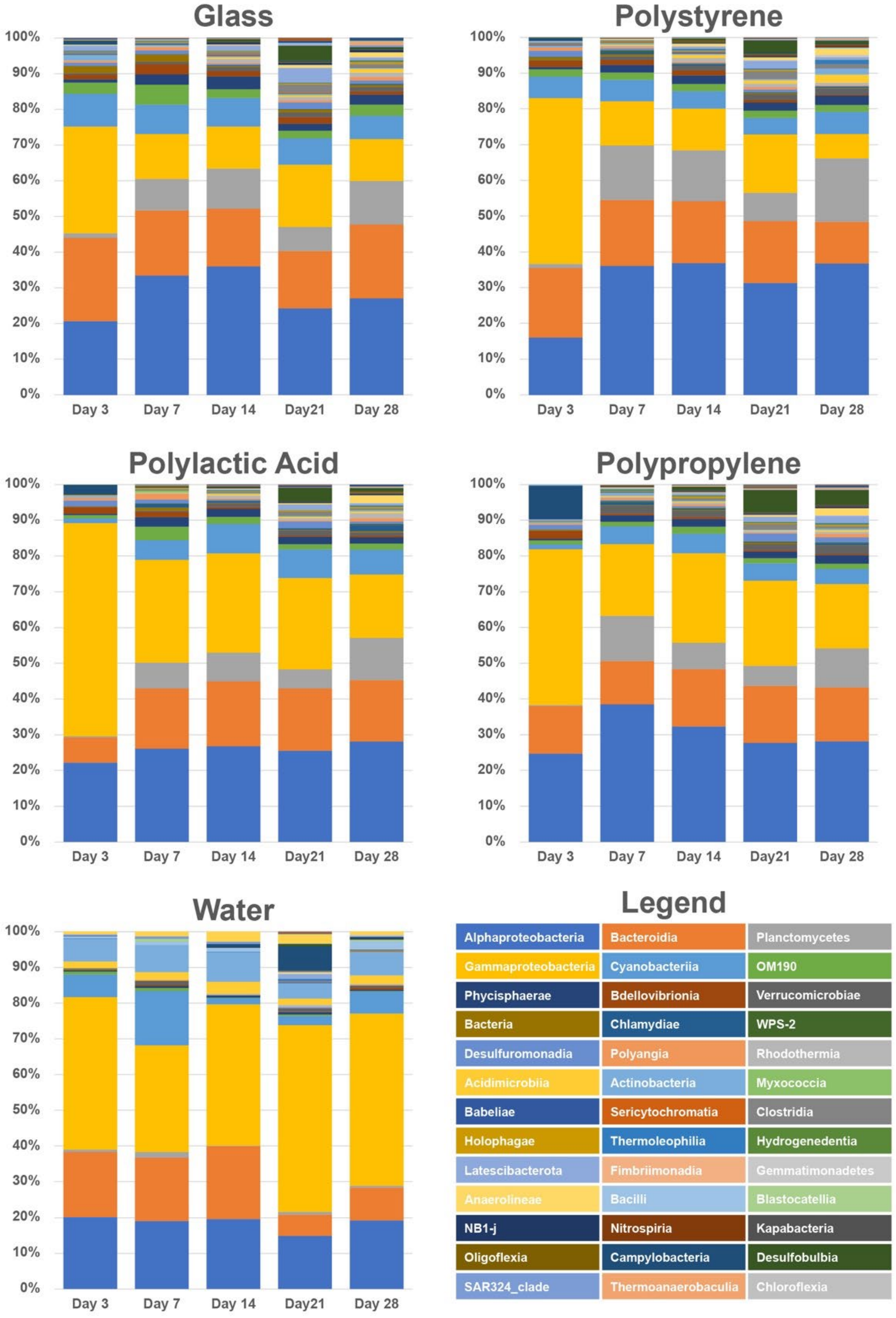

Figure S1. Relative abundance of major bacterial and archaeal lineages at the class level. The relative abundance was analyzed based on the 16S ribosomal RNA gene sequences obtained on day 3, 7, 14, 21 and 28.

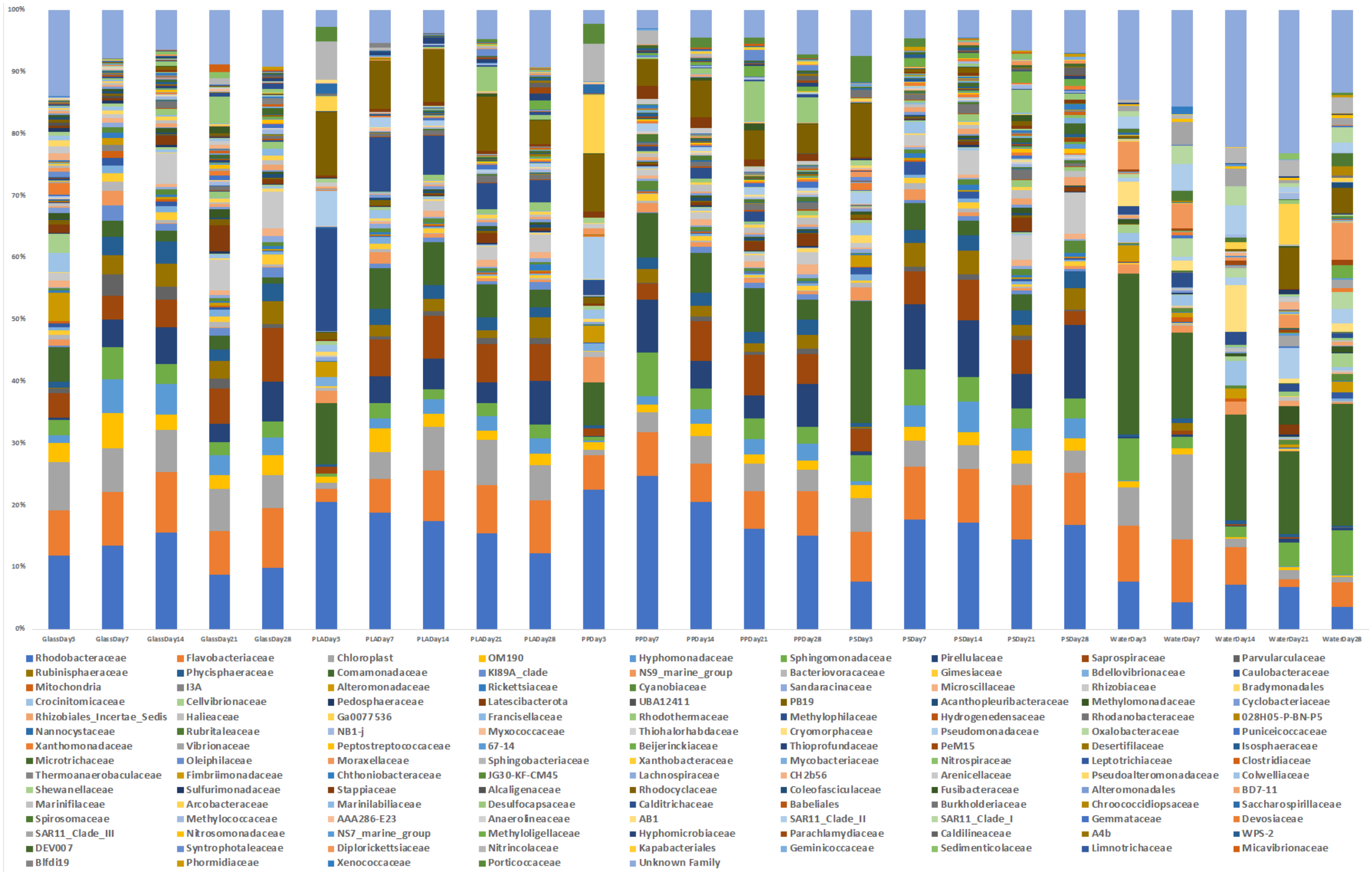

Figure S2. Relative abundance of major bacterial and archaeal lineages at the family level. The relative abundance was analyzed based on the 16S ribosomal RNA gene sequences obtained on day 3, 7, 14, 21 and 28.
